# Weakly Supervised SVM-Enhanced SAM Pipeline for Stone-by-Stone Segmentation of the Masonry of the Loire Valley Castles

**DOI:** 10.3390/jimaging10060148

**Published:** 2024-06-19

**Authors:** Stuardo Lucho, Sylvie Treuillet, Xavier Desquesnes, Remy Leconge, Xavier Brunetaud

**Affiliations:** 1 Laboratoire PRISME, Université d’Orléans, 45100 Orléans, France; cesar-stuardo.lucho-romero@univ-orleans.fr (S.L.); xavier.desquesnes@univ-orleans.fr (X.D.); remy.leconge@univ-orleans.fr (R.L.); 2 LaMé, Université d’Orléans, 45100 Orléans, France; xavier.brunetaud@univ-orleans.fr

**Keywords:** SAM, SVM, stone, segmentation, masonry

## Abstract

The preservation of historical monuments presents a formidable challenge, particularly in monitoring the deterioration of building materials over time. Chateau de Chambord’s facade suffers from common issues such as flaking and spalling, which require meticulous stone and joint mapping from experts manually for restoration efforts. Advancements in computer vision have allowed machine-learning models to help in the automatic segmentation process. In this research, a custom architecture defined as SAM-SVM is proposed, to perform stone segmentation, based on the Segment Anything Model (SAM) and Support Vector Machines (SVM). By exploiting the zero-shot learning capabilities of SAM and its customizable input parameters, we obtain segmentation mask for stones and joints, which are then classified using SVM. Two more SAMs (three in total) are used, depending on how many stones are left to segment. Through extensive experimentation and evaluation, supported by computer vision methods, the proposed architecture achieves a Dice coefficient of 85%. Our results highlight the potential of SAM in cultural heritage conservation, providing a scalable and efficient solution for stone segmentation in historic monuments. This research contributes valuable insights and methodologies to the ongoing conservation efforts of Château de Chambord and could be extrapolated to other monuments.

## 1. Introduction

The preservation of cultural heritage is a key factor in human history, as it serves as a link to our past, which allows us to understand and appreciate our history, traditions, and considerations of previous generations [1]. Among several types of cultural heritage (CH), the preservation of historical monuments and historic buildings is a wildly interdisciplinary studied field, and many authors use the Structural Health Monitoring (SHM) framework as a damage detection strategy [2] to support preservation efforts. As defined by Bassoli [3], the first step is the characterization of existing civil structures such as stones, columns, doors, and windows, among others, for documentation and damage detection. When conservators or researchers want to develop hypotheses for diagnosing the state of health of a monument’s stonework, they need to create a synthesis of conservation data [4,5,6]. These data may include the provenance of the stone, the types of stone and associated properties, the date of (re)installation, the current state of deterioration, and previous and current treatments, among others. Until now, the most versatile medium used to organize this information has been based on Ortho projection, mainly orthophotos. However, the orthophoto itself may not be a sufficient medium. As most of the available data are related to stones, these need to be segmented for optimal data indexing. The next step is to create a database at the stone level, using specialized software such Qgis, so that quantitative and statistical analyses can be carried out. This is a tedious and time-consuming task conducted by experts.

**Figure 1:**
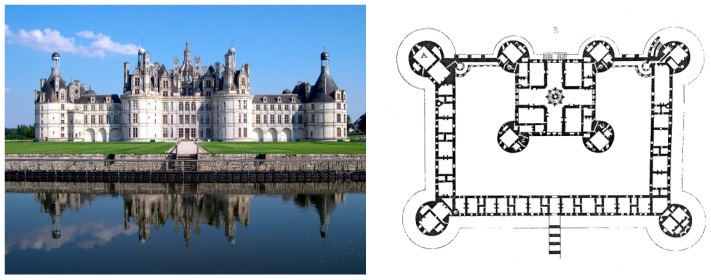
General view of Château de Chambord by GIRAUD Patrick and its plan by Eugène Viollet-le-Duc (via Wikimedia Commons).

This article focuses on automating this process using image segmentation to help experts in this task for monitoring the renaissance castles of the Loire Valley in France in the continuation of previous work [7,8,9]. The most emblematic of these castles is Château de Chambord (Figure 1). It is famous for the characteristic geometry of its architecture, with four massive towers and a double-helix staircase attributed to Leonardo da Vinci, a masterpiece of renaissance engineering. Its construction began in 1519 by King Francis I and took almost 28 years to be completed. Like most renaissance castles, Château de Chambord is built primarily of tuffeau, a soft and porous limestone native to the valley along the Loire River. The color of this stone is creamy white, and its tenderness has enabled craftsmen to create smooth facade walls with almost invisible tone-on-tone joints, making it difficult to segment the stone. This soft stone deteriorates over time due to many environmental factors, with two main types of damage: flaking and spalling [8].

In recent years, a combination of computer vision algorithms and machine-learning techniques have been proposed to aid in stone-by-stone segmentation [10]. In [11], two deep-learning models (SegNet and DeepLab v3+) were tested against traditional edge detection and thresholding methods, in images extracted from orthophotos of the façades of a French renaissance castle built in tufa limestone. The dataset includes 245 images of 256 × 256 px manually labeled for training and testing. In another context, for bricks segmentation, Kajatin [12] proposed the analysis and fusion of eight classifiers (kNN, Bayes, QDA, SVM, decision tree, random forest, AdaBoost, U-Net) for the segmentation of closed range photos of reddish bricks. The dataset used was composed of 27 manually labeled photos of 848 × 480 px. Previously, U-Net-based delineation with Watershed segmentation was used by Ibrahim [13] for two types of bricks (rubble and ashlar masonry) on a dataset of 162 manually labeled images of 512 × 512 px. Loverdos [14] tested five convolutional neural networks (U-Net, U-Net-SM, LinkNet-SM, FPN-SM, and DeepLab V3+) with different configurations (transfer learning, backbone, optimizer, and loss functions) to identify which performs better in the semantic segmentation of brickwork structures. The dataset was comprised of 2814 crops of 224 × 224 px from 107 images with a variety of brick colors, angles, illumination, and resolution. The joints of the brick walls studied in the previous work [12,13,14] are clearly distinguishable from bricks, with distinguishing colors, unlike the limestone facades of renaissance castles used in the present study, which have very homogeneous joints and stones.

A new model, called the Segment Anything Model (SAM), was released in early 2023 by the Meta Research Team [15]. Trained on over a billion semantic masks from open-world images, this model is available to perform a zero-shot learning segmentation of any object of interest in images generically across all application domains (i.e., without additional training). In Cultural Heritage fields, Réby [16] used SAM in a pipeline for labeling objects in a 3D points cloud of Notre Dame Cathedral in a semi-automatic way. On a set of photogrammetric scenes covering different parts of the cathedral, the experts delimited the large objects using 2D bounding boxes. Once the segmentation was obtained by SAM, it was labeled and propagated on the 3D points cloud. This labeling missed the low-level details such stones, windows, columns, etc. Kutlu [17] compared the threshold-based method (TBM), color-based method (CBM), U-Net, SAM, GCoNet+, and UFO-Net for semantics segmentation in masks generated by a multi-view stereo (MVS) scanning process to reconstruct the original object. UFO attained the best results with the highest stability followed by GCoNet, and SAM showed interesting results that needed further improvements.

**Figure 2:**
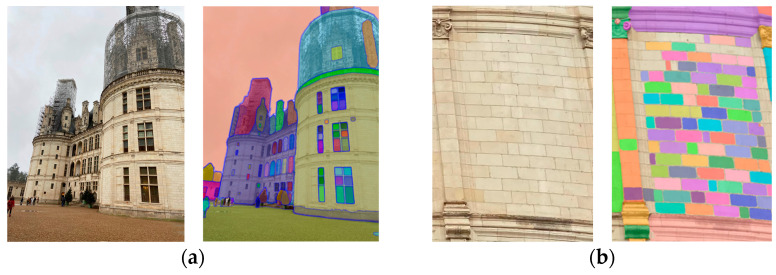
SAM results illustration on two images: (**a**) photo of the South internal façade of Château de Chambord captured with an iPhone 11 (3024 × 4032 px, Apple Inc, California, United States); (**b**) a section of the right central part cropped from the same photo.

We tested SAM on photos of Château de Chambord. Two examples are shown in Figure 2. SAM provides overlapping colored segmentation masks. Depending on how the image is framed, more or less detail is obtained for stone-by-stone segmentation.

Based on these preliminary results, and in the related work reviewed, this article proposes to investigate whether SAM could be an interesting solution to aid stone-by-stone segmentation in historical monuments. SAM is an elegant solution for dispensing with the tedious task of labeling databases to learn the ground truth. On the other hand, the masks generated by SAM cannot be used directly; pre- and postprocessing appear to be necessary. We therefore propose a pipeline exploiting SAM iteratively with a weakly supervised SVM approach. In this paper, the new pipeline proposed based on SAM is compared to the previously tested DL methods in [11] and one more, SegFormerB5, on the same dataset for stone-by-stone segmentation of the masonry of the Loire valley castles. The main interest of using SAM is that it uses zero-shot learning, i.e., it does not require training on a large dataset, and the following step of mask selection is performed with weakly supervised SVM. The rest of the paper is organized as follows: Section 2 includes the dataset composition and the proposed methodology, Section 3 the results and discussion, and finally Section 4 the conclusions.

## 2. Materials and Methods

### 2.1. Dataset

**Figure 3:**
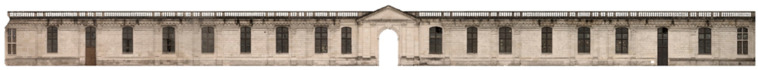
Orthomosaic map of the south façade of Château de Chambord (41,864 × 3828 px), with a resolution of 5 mm^2^ per pixel.

**Figure 4:**
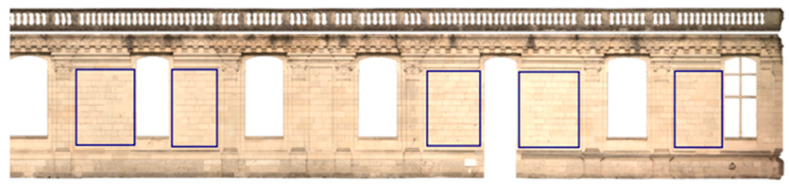
Samples cropped from the south façade of Château de Chambord.

**Figure 5:**
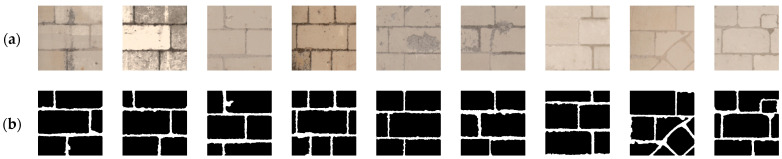
Examples from the stone-by-stone dataset used in [11]: (**a**) 256 × 256 px images cropped from orthomosaic; (**b**) hand-segmented ground truth.

An orthomosaic map of the south facade of Château de Chambord was generated using photogrammetry from 109 photos, as shown in Figure 3, with a resolution of 5 mm^2^ per pixel and a total of 41,864 × 3828 px. From this orthomosaic map, five sections of wall were cropped (as shown in Figure 4) and the joints were carefully segmented by hand to create 245 annotated images with ground truth (256 × 256 px each). The images in the dataset represent crops from the walls where the stones appear throughout all the images, and the labeled joints account for 2.4% of pixels, compared with 97.6% of pixels for stones. Some examples are shown in Figure 5. The dataset is the same as used in [11], where data augmentation (brightness, contrast flipping, and blurring) was used to increase the number of images from 245 to 1715 images for deep learning. 

### 2.2. Segment Anything Model (SAM)

**Figure 6:**
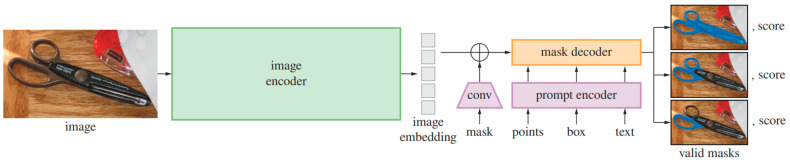
SAM architecture (from [15]).

In 2023, the Segment Anything Model (SAM) was introduced by the Fundamental AI Research (FAIR) team [15] as a new foundational zero-shot inference (or zero-shot learning) model for general-purpose object segmentation in computer vision. At output, SAM delivers a series of overlapping binary masks that can “cut out” any object, of any item in the image. Multiple options are available for automatically generating segmentation masks based on key points (with positive or negative labels, SAM infers the area to be segmented), bounding boxes (SAM segments the bounded object), or both to improve the performance. The model has been trained on the SA-1B dataset, which contains 11 million images (3300 × 4920 px) and 1.1 billion segmentation masks. The SAM architecture is shown in Figure 6. It comprises 3 main components: an encoder including pre-trained vision transformer architecture (ViT) that outputs an image embedding; a prompt encoder that can take points, boxes, text, or masks as inputs; and finally, a mask decoder that maps the image embedding and the prompts to generate output masks probabilities for each location. There are three possible options to be used as an encoder: ViT-B, ViT-L, and ViT-H with a different number of parameters: 91 million for ViT-B, 308 million for ViT-L, and 636 million for ViT-H, and consequently an inference speed that depends on the chosen encoder. SAM also has a set of customizable input parameters, as well as input prompts, which together allow us to obtain more precise segmentation masks framed in a particular context.

**Figure 7:**
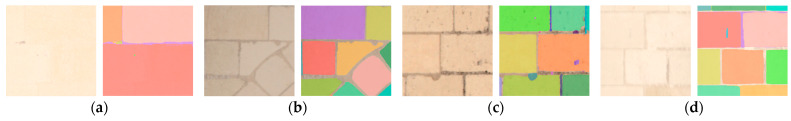
Some examples of masks predicted by SAM on our database with default parameters and ViT-H as the encoder: (**a**) high-brightness image; (**b**,**c**) correct or slightly dark image; (**d**) blurred image.

**Figure 8:**
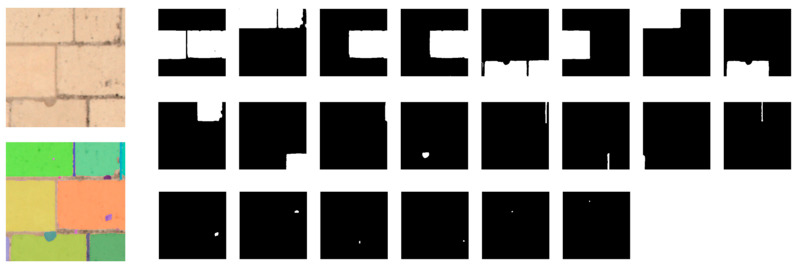
Inferred masks by SAM for Figure 7c.

To see how the SAM model performs in “auto-segmentation” on our dataset, we carried out a few experiments using the open-source Python implementation of SAM [18]. Some results are shown in Figure 7 and Figure 8, using all default parameters and ViT-H as the encoder. The calculation time for all individual masks for each image of 256 × 256 px is about 3–4 seconds with a PC setup using Python 3.9 with Ubuntu 20.04, Intel^®^ Xeon(R) Silver 4208 CPU @ 2.10 GHz × 32 cores, GPU Nvidia RTX A4000 16 GB, and 62.5 GB of memory.

These results call for two remarks:

- As SAM is not particularly trained to segment joints and stone, the predicted masks include both, as shown in Figure 7 and Figure 8, which is correct for SAM but not what we would like for stone-by-stone segmentation;
- Although 3 to 4 seconds may seem short, we are processing a small image of 256 × 256 px, and the total processing time would be considerably longer if applied to a large orthophoto of 41,864 × 3828 px.

Based on these remarks, we found an opportunity to improve the quality of the predicted masks and reduce processing time, using SAM with tunable parameters.

### 2.3. Customizable SAM Parameters

**Figure 9:**
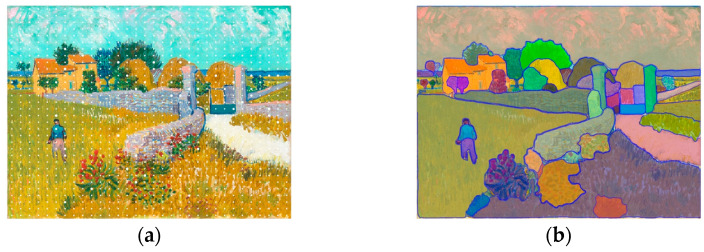
Example of SAM auto-segmentation from [19]: (**a**) grid of key points (white dots), (**b**) generated masks.

The Python implementation of SAM available in open source includes several tunable parameters that control the mask generation [18]. One of the most influential parameter is the number of points_per_side. As shown in Figure 9, the automatic segmentation function predicts masks based on a grid of regularly spaced key points in the image, with a total number of points_per_side^2^. This parameter allows us to control how densely the key points are sampled and consequently the detail in mask generation: the higher the value, the more detailed the mask segmentation (the default grid is 32 points by 32 points).

**Figure 10:**
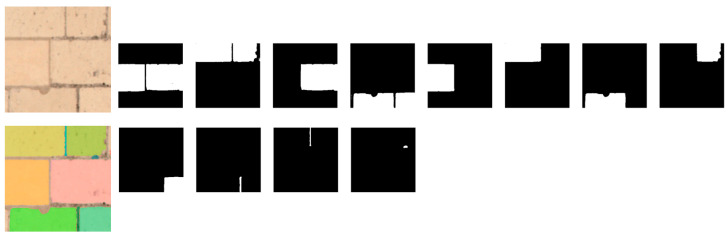
Inferred masks by SAM for Figure 7c with points_per_side reduced to 8.

Different combinations of SAM input parameters were tested and the best configuration that achieved a good balance between performance and time consumption was found by fixing points_per_side to 8. As shown in Figure 10, the number of masks generated from the same image as Figure 8 is reduced to 12 instead of 22 as previously (with a default value of 32 points_per_side), and the processing time is now between 1 and 1.2 s.

Some generated masks now cover the stones well, but there are still some overlapping masks to be eliminated; the remaining masks also contain joints or holes, as shown in Figure 10. SAM provides various data on the masks generated for postprocessing, such as the area, bounding box coordinates, prediction quality (IoU), stability score, etc., that could be used for filtering, but threshold values are not easy to define arbitrarily and may lack generality.

The next section proposes a pipeline exploiting SAM iteratively with a weakly supervised approach to improve stone segmentation. 

### 2.4. Proposed Pipeline

**Figure 11:**
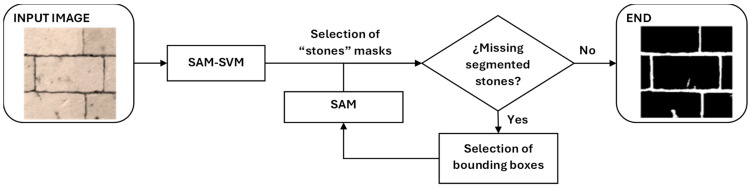
Proposed pipeline.

In order to perform stone-by-stone segmentation on limestone masonry images, the pipeline shown in Figure 11 is proposed. There are two main innovations: firstly, a weakly supervised SVM classifier is introduced to label the masks generated by SAM in the first inference, then SAM is applied iteratively using bounding boxes as input prompts to refine segmentation and generate the best masks on all stones.

#### 2.4.1. SVM-Enhanced SAM for Mask Filtering

As shown in Figure 10, the masks generated in the first instance by SAM from a grid of key points, with the density (point_per_side) reduced to 8, include not only stones, but also joints, holes, and interfering areas. The aim is to automatically label the generated masks into different categories using a SVM classifier. SVM or Support Vector Machines is a supervised learning algorithm used mainly for classification but can also be used for regression analysis and outliers detection [20]. To classify, it uses a hyperplane (or a set of hyperplanes in infinite-dimensional space) that separates the data into different classes, where the maximum distance between this hyperplane and the closest data points is known as support vectors. If the data cannot be divided linearly, a separator is added, and all the data are transformed so that the hyperplane clearly divides the classes. The function used to transform the data is also known as the kernel, the most common being linear kernel, polynomial kernel, Radial Basis Function (RBF), and sigmoid kernel.

**Figure 12:**
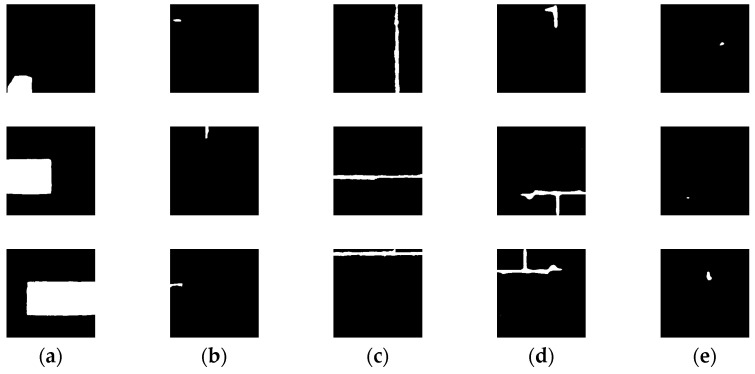
Some samples of SAM masks used for SVM training. (**a**) Stones; (**b**) Half-lines; (**c**) Full lines; (**d**) Perpendicular lines; (**e**) Holes.

**Figure 13:**
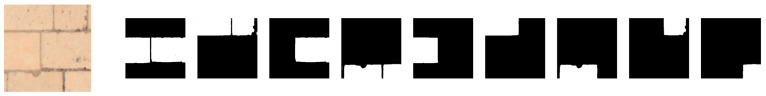
Masks assigned to the label “stone” by the SVM classifier among the output masks of the image shown in Figure 10.

To train the SVM classifier to be included in the proposed architecture, 30 images were randomly selected from the dataset, then SAM was applied in the default configuration to automatically generate a total of 416 masks. These masks were manually labeled into 5 classes, as shown in Table 1, with the number of masks generated for each class. An example of each class is shown in Figure 12. For the full and perpendicular lines classes, there were not many masks for training, so data augmentation (rotation and mirroring) was used. The trained SVM model had an accuracy of 83.3% for the stone class. As an example, the SVM classifier gives the “stone” label to 9 masks out of the 12 on the output masks of the image presented in Figure 10; these “stone” masks are shown in Figure 13. 

#### 2.4.2. Missing Segmented Stones Test

**Figure 14:**
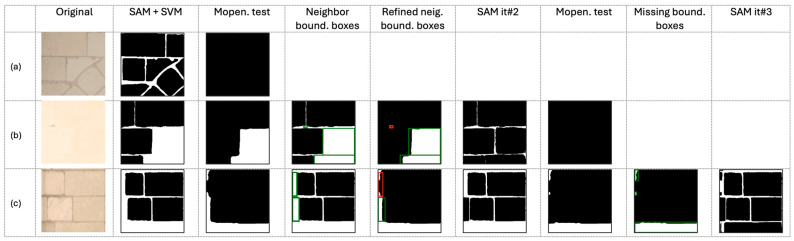
Three examples of images through the proposed pipeline: (**a**) segmentation is optimal after the first SAM inference and mask filtering by the SVM classifier; (**b**) after the second SAM iteration, the image is fully segmented; (**c**) the image needed three SAM iterations to complete segmentation.

To check if all the stones are segmented, a morphological opening is performed on the mask after a bitwise inversion (stone in black) with a disk-shaped footprint of radius 10. If more than 1% of white pixels are left after the opening, then the original image will go through a second iteration of SAM. Figure 14 shows three examples of images processed through the proposed pipeline. After the first SAM inference and mask filtering by the SVM classifier, the three images present different situations: (**a**) the first-row image is optimally segmented, and the percentage of white pixels remaining after the morphological opening operation is 0%; (**b**) the second-row image presents missing stones with 27.81% white pixels remaining after the opening operation; (**c**) the third-row image has missing stones with 17,78% white pixels remaining after the opening operation. For the latter two, the morphological filter test is not conclusive, and the segmentation process continues with further SAM iterations.

#### 2.4.3. Iterations of SAM with Bounding Boxes as Inputs

If the morphological filter test is not conclusive, SAM is repeated on the problematic bounding boxes. SAM performs better segmentation by using bounding boxes as input prompts, and the processing time decreases as the segmentation is carried out only in the input prompt boxes. To generate the input prompt bounding boxes, all the contours of the detected stones are identified from the masks, then using the height of each contour, a horizontal neighbor bounding box is identified. SAM is applied only for the boxes that overlap the white pixels’ mask over 30%. The two last SAM iterations based on the bounding box complete the stone segmentation differently. The 2nd iteration appends stones on the same horizontal line on the left or on the right, and the 3rd iteration supplements all the missing stones in the image by creating bounding boxes for the remaining white areas. As shown in Figure 14 (column SAM it#2), the segmentation of Figure 14b,c is well refined: Figure 14b is fully segmented with a percentage of white pixels remaining of 0%, while Figure 14c presents missing stones with a percentage of white pixels up to 14.94% after morphological opening. In this case, a third SAM iteration is required.

## 3. Results and Discussion

The proposed pipeline was applied to the dataset presented in Section 2.1 and compare to other state-of-the-art solutions for stone-by-stone segmentation. Following the guidelines given in [11], SegNet and DeeplabV3+ were finetuned by transfer learning, and tested; in the same way, SegFormer-B5 [21] was also trained and tested. From the dataset, 15% (257 images) were used for testing and 85% for training the deep-learning architectures (SegNet, DeeplabV3+, SegFormer). Finally, a default SAM version was tested (keeping default parameters) and performance evaluated by merging all the predicted masks.

All the results are shown in Table 2. The processing time of the different steps of the proposed pipeline is also analyzed in Table 3. The experiments were carried out on a PC setup (Intel^®^ Xeon€ Silver 4208 CPU @ 2.10 GHz × 32 cores, GPU Nvidia RTX A4000 16 GB, 62.5 GB of memory).

**Figure 15:**
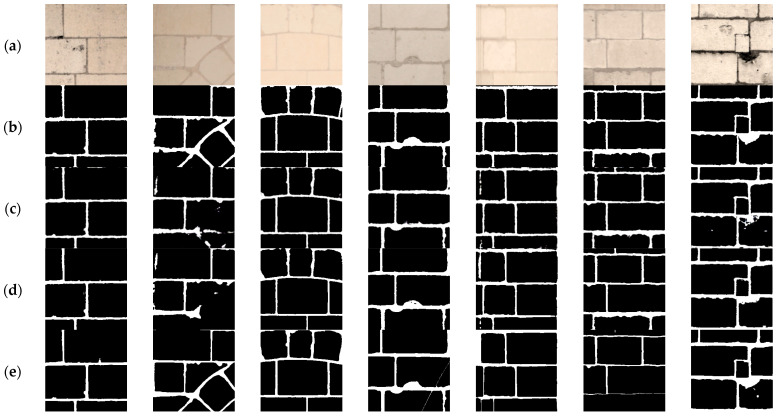
Some examples of results obtained. (**a**) Original image; (**b**) Ground truth; (**c**) DeepLabV3+; (**d**) SegFormerB5; (**e**) SAM-SVM.

The Dice values achieved are up to 85% which is really promising, taking into consideration that no training has been conducted for SAM based on stone segmentation. Furthermore, the number of sampled points was decreased to eight in SAM to attain less information on joints but more information on the “big” stones in the photos, and this decrease in the sample points is compensated by using SAM three times (depending on the image) along with SVM. Some qualitative results can be seen in Figure 15.

As shown in Table 2, SegFormerB5 had the best result among all the tested algorithms, even better than the proposed architecture. However, SegFormerB5 was trained on 1458 images, while SAM-SVM had a small training set only for the SVM block on the outputs mask of SAM itself, making the 85% Dice obtained a promising result.

As shown in Table 3, the time for some images is almost 3 seconds in some cases, which resembles the time SAM took with all its default parameters; however, read and write image operations are also involved in the whole pipeline, and this is one of the reasons why the time has risen from 1 second up to 3, but this could be improved to decrease the time.

The processing chain proposed for the selection of relevant masks provided by SAM can be applied to all walls in any castles in the Loire Valley of renaissance style, and it can also be generalized to other scenarios, taking into consideration that SVM should be trained on which classes to reject or retain.

## 4. Conclusions

This paper proposes a novel pipeline composed of existing algorithms like SAM and SVM for limestone stone segmentation in Loire Valleys châteaux. The combination of weakly supervised SVM along with SAM improves the zero-shot learning for stone-by-stone segmentation and reduction in processing time. Overall, with the presented pipeline, we achieved 85% of Dice with an average processing time of 1.81 per image (256 × 256 px), which is better than SegNet (83%), trained on a stone dataset, but inferior to DeepLab V3+ and SegFormer. 

By looking at the individual results, SAM performed better than SegFormer and DeepLab V3+ in images with diagonal stones and attained really good results depending on the illumination and contrast of the image, as shown in some results in Figure 15.

Based on the experiments performed with SAM to achieve a good stone-by-stone segmentation, a postprocessing of the output mask is necessary in order to achieve results that are useful for cultural heritage. Likewise, the SAM tunable parameters play a vital role depending on the image resolution and on the size the of objects that are being looked at, such as big stones in small photos (this research) or a group of stones in a complete wall.

For orthophotos with other elements such windows, moldings, and doors, SAM could provide an initial segmentation of objects, and then SVM could be trained on these new masks generated by SAM to keep only stones and aid the final mask for stone-by-stone segmentation.

## Figures and Tables

**Table 1:** Training set for SVM classifier by class label.

#	Class	Number of Masks
1	Stones	257
2	Half-lines	33
3	Full lines	18
4	Perpendicular lines	16
5	Holes	92
	Total	416

**Table 2:** Comparative metrics.

	Accuracy	Dice	IoU	Recall	Precision	Avg. Inference Time
SegNet	0.9580	0.8374	0.7236	0.8909	0.7947	0.11 s
DeepLab V3+	0.9764	0.9114	0.8421	0.9227	0.9043	0.16 s
SegFormerB5	0.9800	0.9239	0.8633	0.9418	0.9106	1.45 s
Default SAM	0.9252	0.6721	0.5457	0.8741	0.5915	3.04 s
SAM + SVM	0.9644	0.8589	0.7637	0.9025	0.8342	1.81 s

**Table 3:** Processing time metrics (in seconds).

	Quantity	Max	Min	Avg	Median	Std
All test set	257	3.17	0.94	1.81	1.50	0.54
Images through SAM Iteration 1	152	1.70	0.94	1.38	1.37	0.12
Images through SAM Iterations 1 and 2	101	3.17	1.91	2.39	2.37	0.20
Images through SAM Iterations 1, 2, and 3	4	3.01	2.89	2.96	2.97	0.05

## Data Availability

The data presented in this study are available on request from the corresponding author due to the privacy of the generated dataset.
